# A New Instrument Combines Cognitive and Social Functioning Items for Detecting Mild Cognitive Impairment and Dementia in Parkinson’s Disease

**DOI:** 10.3389/fnagi.2022.913958

**Published:** 2022-06-16

**Authors:** Ya-Wen Yu, Chun-Hsiang Tan, Hui-Chen Su, Chung-Yao Chien, Pi-Shan Sung, Tien-Yu Lin, Tsung-Lin Lee, Rwei-Ling Yu

**Affiliations:** ^1^Institute of Behavioral Medicine, College of Medicine, National Cheng Kung University, Tainan City, Taiwan; ^2^Department of Neurology, Kaohsiung Medical University Hospital, Kaohsiung Medical University, Kaohsiung City, Taiwan; ^3^Graduate Institute of Clinical Medicine, College of Medicine, Kaohsiung Medical University, Kaohsiung City, Taiwan; ^4^Department of Neurology, National Cheng Kung University Hospital, College of Medicine, National Cheng Kung University, Tainan City, Taiwan

**Keywords:** dementia, Montreal Cognitive Assessment, social functioning, Parkinson’s disease, mild cognitive impairment

## Abstract

**Background:**

The commonly used screening tests for Parkinson’s disease (PD) are the Montreal Cognitive Assessment (MoCA) and Mini-Mental State Examination (MMSE), both of which only focus on cognitive function. A composite assessment that considers both cognitive and social dysfunction in PD would be helpful in detecting mild cognitive impairment (MCI) and PD dementia (PDD).

**Objective:**

We aimed to simplify the commonly used tools and combine cognitive and social functioning tests to detect early MCI and PDD.

**Materials and Methods:**

A total of 166 participants (84 PD patients and 82 healthy) were recruited who completed the MMSE, MoCA, PD social functioning scale (PDSFS), clock drawing test, activities of daily living, comprehensive neuropsychological assessment (e.g., executive, attention, language, memory, and visuospatial functions), and movement disorder society (MDS)-unified PD rating scale. According to the MDS diagnostic criteria, the patients were grouped into PD-nonMCI, PD-MCI, or PDD.

**Results:**

To detect PD-MCI, the optimal cut-off scores for the simplified MoCA and the combined test were 9 and 35. The discrimination values measured by the area under the receiver operating characteristic curve (AUC) of the two tests were 0.767 (*p* < 0.001) and 0.790 (*p* < 0.001). When the simplified MoCA was 7 or the combined test 30, the patients would be classified as having PDD. The AUCs of the two tests were 0.846 (*p* < 0.001) and 0.794 (*p* = 0.003).

**Conclusion:**

We suggest considering both cognitive and social functions when detecting PD-MCI and PDD.

## Introduction

Parkinson’s disease (PD) is a chronic, progressive neurodegenerative disorder. The number of individuals diagnosed with PD has grown significantly in the past three decades worldwide (Armstrong and Okun, 2020). Recent global data indicate that the prevalence of PD in ages 40–49 is 41 per 100,000 individuals and up to 1903 per 100,000 individuals among those over the age of 80 years (Pringsheim et al., 2014). Motor symptoms are the predominant clinical manifestation of PD, but non-motor symptoms are also prevalent (Liu et al., 2015; Schapira et al., 2017), such as cognitive dysfunction (Yu et al., 2010, 2012a, 2015b), social brain dysfunction (Yu et al., 2012b, 2018; Yu and Wu, 2013a), and sleep disturbances (Yu et al., 2015a). Cognitive dysfunction may affect how patients effectively deal with real-life problems, emphasizing an individual’s function (Anderson et al., 2013). A systematic review yielded a PD patient with dementia (PDD) point prevalence of 31.3% (Aarsland and Kurz, 2010), and the cumulative prevalence from 8 years of follow-up found that up to 78% of PD patients eventually developed PDD (Aarsland et al., 2003). Mild cognitive impairment (MCI) may be a precursor of PDD (Goldman and Sieg, 2020) and is common in PD patients without dementia (mean cross-sectional prevalence, 26.7%; range, 18.9–38.2%) (Litvan et al., 2011), and can be present in patients with early-stage PD. A meta-analysis indicates that 25% of patients with PD with normal cognition progressed to PD with MCI (PD-MCI), and 20% of PD-MCI progressed to PDD within 3 years (Saredakis et al., 2019).

The clinical diagnostic criteria for PDD published by the movement disorder society (MDS) task force include the following core features: impairment in more than one cognitive domain, representing a decline from premorbid level, and deficits severe enough to impair an individual’s function in daily life (e.g., social, occupational, or personal care) (Dubois et al., 2007; Emre et al., 2007). Patients’ cognitive function can be measured through cognitive tests, such as the Mini-mental status examination (MMSE) (Folstein et al., 1975) and Montreal Cognitive Assessment (MoCA) (Nasreddine et al., 2005). However, most patients with PD have retired and have no occupational function performance that could be used as a basis. Therefore, the social function has become an essential aspect of determining whether a person has entered a stage of dementia or not. Although “social function” is a crucial component in determining an individual’s function and it is a less-noticed aspect that determines the quality of life (Bettencourt and Sheldon, 2001; Perepezko et al., 2019). Human beings have social lives, and the connection between self and society is crucial, especially for patients (Yu and Wu, 2013b). Changes in social role functioning impede individual wellbeing and quality of life (Bettencourt and Sheldon, 2001; Yu and Wu, 2013b; Perepezko et al., 2019). In addition, social function deficits escalate a person’s risk of dementia (Fankhauser et al., 2015) and expedite the dementia process (Bennett et al., 2006). It has always been difficult for clinicians to evaluate patients’ social functioning. The PD social functioning scale (PDSFS) has been developed to specifically and precisely measure PD patients’ social functioning (Su et al., 2020). However, this field needs more empirical evidence for clinical applications in determining PD-MCI or PDD.

The MMSE (Folstein et al., 1975) and MoCA (Nasreddine et al., 2005) are currently commonly used as general cognitive screening tools in clinical practice (Bezdicek et al., 2020; Yu et al., 2020). The original MMSE and MoCA accurately differentiate cognitive impairment (MCI or Alzheimer’s disease) from normal cognitive aging (Pinto et al., 2019). The original MoCA has adequate psychometric properties as a screening instrument for detecting MCI or dementia in patients with PD (Hoops et al., 2009; Dalrymple-Alford et al., 2010; Robben et al., 2010; Marras et al., 2013; Biundo et al., 2014; Kandiah et al., 2014; Ozdilek and Kenangil, 2014; Xu et al., 2015; Uysal-Cantürk et al., 2018; Badrkhahan et al., 2019; Bezdicek et al., 2020; Mazancova et al., 2020), and the optimal score for detecting was explored (Hoops et al., 2009; Dalrymple-Alford et al., 2010; Robben et al., 2010; Chen et al., 2013; Marras et al., 2013; Biundo et al., 2014; Kandiah et al., 2014; Ozdilek and Kenangil, 2014; Federico et al., 2015; Xu et al., 2015; Uysal-Cantürk et al., 2018; Badrkhahan et al., 2019; Mazancova et al., 2020; Yu et al., 2020; Table 1).

**TABLE 1:** Studies assessing cognitive function by MMSE and MoCA in patients with Parkinson’s disease.

Study	Demographic data						Criteria	Result
	Language	Sample	Age	Male (%)	Education	H & Y		
Hoops et al., 2009	English	PD-N:92 MCI or PDD: 40	PD-N: 63.9 MCI or PDD: 68.1	PD-N:72.8% MCI or PDD: 82.5%	PD-N: 16.5 MCI or PDD: 16.2	-	MCI: Winblad et al. (2004) criteria PDD: MDS Level I	MoCA to detect MCI is 27, PDD is 25. MMSE to detect MCI is 30, PDD is 29. MoCA was superior to MMSE.
Dalrymple-Alford et al., 2010	English	HC: 47 PD: 114	HC: 67.3 PD-N: 64.5 MCI: 71.5 PDD: 73.4	HC: 66.0% PD-N:69.4% MCI: 71.4% PDD: 85.7%	HC: 13.7 PD-N: 13.2 MCI: 12.3 PDD: 12.9	PD-N: 13.2 MCI: 12.3 PDD: 12.9	MCI: NPT < 1.5 SD PDD: MDS Level II	MoCA to detect MCI is 26, PDD is 21. MMSE to detect MCI is 29, PDD is 27. MoCA was superior to MMSE.
Robben et al., 2010	Dutch	PD: 41	PD-Y: 58.8 PD-O: 78.5	PD-Y:59.0% PD-O:63.2%	PD-Y: 6.8 PD-O: 9.9	2.0^a^	According to a neuropsychologist to diagnose the patient as cognitively impaired or not.	MoCA to detect PD-Y (<65 years) is 23, and PD-O is 22.
Chen et al., 2013	Chinese	HC: 85 PD: 616	PD-ND: 66 PDD: 68	PD-ND:64.5% PDD: 57.7%	PD-ND: 12 PDD: 9	-	PDD: MDS Level I	MoCA to detect PDD is 23.
Marras et al., 2013	English	PD: 139	MCI: 71.1 nMCI: 71.1	MCI: 63% nMCI: 69%	MCI: 15.3 nMCI: 16.1	-	MCI: MDS Level II	MoCA to detect MCI is 26. MMSE is unable to detect MCI.
Biundo et al., 2014	Italian	PD: 105	PD-N: 58.4 MCI: 66.8 PDD: 71.2	PD-N:37.8% MCI: 30.6% PDD: 18.8%	PD-N: 12.1 MCI: 10.3 PDD: 10.9	-	MCI: MDS Level II PDD: MDS Level I	MoCA to discriminate PD-N from MCI is 26, to distinguish MCI from PDD is 20. MMSE to discriminate MCI from PDD is 25. MoCA was more sensitive than MMSE to detect MCI but not PDD.
Kandiah et al., 2014	English	PD: 95	PD-N: 64.3 MCI: 70.5	PD-N: 77% MCI: 58.8%	PD-N: 10.9 MCI: 9.29	PD-N: 1.95 MCI: 2.07	MCI: MDS Level II PDD: MDS Level I	MoCA to detect MCI is 26.
Ozdilek and Kenangil, 2014	Turkish	HC: 50 PD: 50	HC: 63.3 PD-N: 58.3 MCI: 63.3 PDD: 67.4	HC: 44% PD-N: 64% MCI: 77% PDD: 67%	HC: 10.0 PD-N: 10.0 MCI: 7.3 PDD: 5.6	-	MCI: (Petersen et al., 2001; Winblad et al., 2004) criteria PDD: two NPT < 1.5 SD and impaired instrumental activities of daily living.	MoCA to detect MCI is 21. MMSE to detect MCI is 26. MoCA is suitable to detect MCI, MMSE is the instrument of choice to detect PDD.
Xu et al., 2015	Chinese	PD:140	MCI: 68.7 PD-N: 68.0	MCI: 57.7% PD-N:55.1%	MCI: 9.9 PD-N: 10.1	MCI: 2.6 PD-N: 2.3	MCI: cognitive decline, at least 1 NPT impaired, preserved daily function, not dementia.	MoCA to detect MCI is 26.
Federico et al., 2015	Italian	PD: 43	nMCI: 67.5 MCI: 68.9	nMCI: 57% MCI: 68%	nMCI: 8.7 MCI: 8.3	nMCI: 1.9 MCI: 2.5	MCI: MDS Level II	MoCA to detect MCI is 25; MMSE is 30. MMSE was superior to MoCA.
Uysal-Cantürk et al., 2018	Turkish	PD: 68	PD-N: 58.9 MCI: 63.6	PD-N:66.7% MCI: 33.0%	PD-N: 8.44 MCI: 8.76	-	MCI: MDS Level II	MoCA to detect MCI is 24; MMSE is 29. The ability to detect PD-MCI in MMSE and MoCA was similar.
Badrkhahan et al., 2019	Persian	PD: 73	PD-N: 69.5 MCI: 73.5 PDD: 78.5	PD-N:56.7% MCI: 55.6% PDD: 70.0%	PD-N: 12^a^ MCI: 13^a^ PDD: 12^a^	-	-	MoCA to discriminate NC from MCI is 24, to distinguish MCI from PDD is 19.
Mazancova et al., 2020	Malayalam	PD: 141	-	-	-	-	-	MoCA to discriminate NC from MCI is 25.
Yu et al., 2020	Chinese	PD: 168	CI: 66.47 MCI: 71.96	CI: 45.92% MCI:54.92%	CI: 12.07 MCI: 7.91	CI: 2.14 MCI: 2.16	MCI: MDS Level II	MoCA to discriminate CI from MCI is 21, MMSE is 25. MMSE and MoCA are suitable for the detection of cognitive dysfunction in PD.

*HC, healthy controls; PD, Parkinson’s disease; PD-ND, non-demented PD; MCI, PD with mild cognitive impairment; PDD, PD with dementia; PD-Y, young group of PD; PD-O, old group of PD; nMCI, PD without mild cognitive impairment; PD-N, PD with normal cognition; CI, PD cognitively intact; H & Y, Hoehn–Yahr stage; NPT, neuropsychological test; MDS, movement disorder society.*

*^a^Value calculated by the median.*

Most studies found that it has superior psychometric properties to the MMSE (Hoops et al., 2009; Dalrymple-Alford et al., 2010; Marras et al., 2013; Kandiah et al., 2014) in detecting a cognitive decline in patients with PD. The MoCA 5-min protocol was established to rapidly screen post-stroke vascular cognitive impairment (Dong et al., 2015) and translated to different languages (e.g., English and Simplified Chinese version) for time efficiency during clinical evaluation. Dong et al. (2015) suggested that the MoCA 5-min was suitable for rapid screening of the cognitive impairment in PD. Other studies have reported a weighted MoCA algorithm (Fengler, 2016) and shorter versions (e.g., English, Czech, and Simplified Chinese version) of MoCA (Dong et al., 2015; Roalf et al., 2016; Bezdicek et al., 2020) to aim explicitly at the PD population. The MoCA algorithm weighted the scores of the visuospatial domain and decreased the proportion of orientation. The Short MoCA-Czech and Short MoCA-US were constructed by the item response theory and computerised adaptive testing analytic techniques. Both the MoCA algorithm and three short versions of MoCA were specific to the neuropathology of PD to discriminate better cognitively intact from cognitively impaired PD patients (Dong et al., 2015; Fengler, 2016; Roalf et al., 2016; Bezdicek et al., 2020). We summarised the content of these short versions of MoCA in Table 2. Moreover, Bezdicek et al. (2020) recently found that languages may affect the short version of MoCA, and they suggested that cultural background and languages should be considered in test development.

**TABLE 2:** Summarize the content of the MoCA algorithm and three short versions of MoCA.

Subtests	MoCA algorithm (Fengler, 2016)	MoCA-5-min (Dong et al., 2015)	Short MoCA-US (Roalf et al., 2016)	Short MoCA-Czech (Bezdicek et al., 2020)
Visuospatial/executive	Y	-	Y	Y
Naming	Y	-	Y	-
Memory	Y	Y	Y	Y
Attention	Y	-	Y	Y
Language	Y	Y	Y	Y
Abstraction	Y	-	Y	Y
Orientation	Y	Y	Y	-

*MoCA, Montreal Cognitive Assessment; Y, keep the subtest; -, delete the subtest.*

Patients’ cognitive function and independent daily life are crucial for diagnosing MCI or dementia. Clinically, physicians often use cognitive tools to examine the cognitive function and experience to determine whether the patients’ social and occupational functions are impaired and further diagnose whether the patients have MCI or dementia. There have been many studies in the past that have analysed and compared various cognitive testing tools for the detecting ability (e.g., MoCA) (Hoops et al., 2009; Dalrymple-Alford et al., 2010; Marras et al., 2013; Biundo et al., 2014; Ozdilek and Kenangil, 2014; Uysal-Cantürk et al., 2018; Badrkhahan et al., 2019; Bezdicek et al., 2020; Mazancova et al., 2020; Yu et al., 2020); moreover, the objective measurement scale for social functioning has just been published (Su et al., 2020). However, to the best of our knowledge, no studies explored the efficacy of combining cognitive and social functioning measures to detect the MCI and dementia in patients with PD. Given that cognitive and social functions are crucial for detecting PD-MCI (Litvan et al., 2011) and PDD (Dubois et al., 2007), we aimed to provide a handy and helpful measurement tool to detect PD-MCI and PDD while considering both cognitive and social functions.

## Materials and Methods

### Participants

One hundred and sixty-six participants were recruited (84 patients with PD and 82 healthy controls, HC) in this study. The outpatients were diagnosed with idiopathic PD by neurologists from teaching hospitals. According to the recommendations of the MDS task group diagnosis criteria for PD with MCI (Litvan et al., 2011) and possible dementia (Dubois et al., 2007), our patients with PD were divided into the following three groups: PD patients without MCI (nonMCI), PD-MCI, and PDD.

The age-matched HC group was recruited from the community. Participants’ inclusion criteria were: basic speaking and reading skills (able to understand after explanation and could provide informed consent), no severe systematic disease, and no consumption of drugs affecting neurocognitive function. Participants were excluded if they had atypical parkinsonism, medical conditions that may cause cognitive dysfunction, and comorbidities, such as hepatitis B, hepatitis C, delirium, head trauma, psychiatric illness (e.g., depressive disorders, anxiety disorders, etc.), and substance use. No HC had a global cognitive problem (i.e., two neuropsychological tests were below one standard deviation).

All participants provided informed consent before participating in the study, and all experiments were conducted per the 1975 Declaration of Helsinki. Additionally, the ethical research committee of the hospitals approved the study protocols.

### Ethical Compliance

The institutional review boards at National Cheng Kung University Hospital and Kaohsiung Medical University Hospital provided formal approval for the study procedures. All participants (or a legally authorised representative) provided written informed consent.

### Measurement

#### Demographic and Clinical Information

All participants underwent a comprehensive clinical evaluation that included collecting information on demographic data, medical history, daily activities appraisal [i.e., Activity of daily living scale (ADLs) (Katz, 1983)], and social functioning assessment [i.e., PDSFS (Su et al., 2020)]. PDSFS is a well-developed tool to measure patients’ social function and provides good reliability (Cronbach’s alpha: 0.883) and convergent and discriminative validities (Su et al., 2020). The scale has three factors, including “Family Life, Hobbies, and Self-Care (FHS),” “Interpersonal Relationship and Recreational Leisure (LRRL),” and “Social Bond (SB)” (Su et al., 2020). Moreover, the levodopa equivalent daily dose (LED) and motor status by the MDS-unified PD rating scale (Yu et al., 2017) of patients with PD were collected.

#### Neuropsychological Assessment

The comprehensive neuropsychological assessment (Table 3) was used to fulfil MDS PD-MCI level II diagnostic criteria and help neurologists classify PD with and without MCI. The MMSE and the original MoCA were useful screening tools to measure patients’ global cognitive function and have good psychometric properties. MMSE has good reliability (test-retest: 0.988) and concurrent validity (Folstein et al., 1975). The original MoCA has good reliability (Cronbach’s alpha: 0.83) and discriminative validities (Nasreddine et al., 2005).

**TABLE 3:** Cognitive domains and neuropsychological tests.

Domain	Neuropsychological tests
Executive function	The number of categories achieved Modified Wisconsin Card Sorting Test (MCST-C) (Nelson, 1976)
	Colour Trails Test part B (CTT-B) (D’Elia et al., 1996)
Attention and working memory	WAIS-III: Digit span (Chen, 2002)
	Colour Trails Test part A (CTT-A) (D’Elia et al., 1996)
Language	WAIS-III: Similarities (Chen, 2002)
	Category fluency (Hua et al., 1997)
Memory	WMS-III: Logical Memory (LM-II) (Hua et al., 2005)
	WMS-III: Visual Reproduction (VR-II) (Hua et al., 2005)
Visuospatial function	WAIS-III: Block design (BD) (Chen, 2002)
	WAIS-III: Matrix Reasoning (MR) (Chen, 2002)

*WAIS-III, Wechsler Adult Intelligence Scale-third edition; WMS-III, Wechsler Memory Scale-third edition.*

### Statistical Analysis

All analyses were performed using SPSS V.17 software (SPSS, Chicago, IL, United States). The level of significance was set at α = 0.05. After accounting for the number of tests performed, we modified the overall alpha criterion for significance. The Bonferroni correction was used to adjust *p* value. Continuous variables were expressed as mean with standard deviation (SD) and categorical variables as percentages. The Kolmogorov–Smirnova test or Shapiro–Wilk test determined if a data set was well-modelled by a normal distribution. The Spearman correlation coefficient was used to evaluate the relationship between different ordinal test measures and avoid collinearity.

First, demographic and disease-related characteristics were summarised with descriptive statistics. Analysis of Covariance (ANCOVA) was used to compare the scores of the MMSE, original MoCA, and neuropsychological tests between the groups with age, sex, and education adjusted. Dunn’s *post hoc* tests were used for *post hoc* comparisons.

Second, the Spearman correlation coefficient was used to evaluate the relationship between variables. Highly correlated (above 0.7) variables were deleted to avoid all input variables having a high degree of collinearity and affecting the model variance. Then, we used logistic regression analysis to explore the domains/factors to achieve the best predictive power, develop simplified versions of MoCA and PDSFS, and combine the two tests. The model specifically predicted the PD-MCI and PDD groups.

Third, the receiver operating characteristic (ROC) curve method was applied to find an optimal cutoff of the simplified versions of MoCA, PDSFS, and a combination of the two tests. In addition, the Wilcoxon test was used to compare two ROC curves (Hanley and McNeil, 1982). Generally, an area under the curve (AUC) of 0.5 suggests no discrimination; 0.7–0.8 is considered acceptable, 0.8–0.9 is deemed excellent, and more than 0.9 is deemed outstanding. The optimal cutoff with the maximum AUC was used to differentiate PD-nonMCI, PD-MCI, or PDD in clinical practice.

## Results

### Demographic and Clinical Characteristics, and Using the Mini-Mental State Examination and Its Subtest to Detect Parkinson’s Disease With Mild Cognitive Impairment

Table 4 illustrated the demographic and clinical characteristics of the study groups. A significant difference was found between the HC and all PD groups in terms of sex and years of education. After controlling the impact of sex and education, significant differences were found in the MMSE and original MoCA scores between the HC and all PD groups (Table 5). Significant differences were found between the HC and the three PD groups (i.e., PD-nonMCI, PD-MCI, PDD) for age, sex, and years of education (Table 4). ANCOVA was conducted to control the demographic impact due to the sex and the education effect (Chen et al., 2021). In all neuropsychological tests, we found significant differences between HC, PD-nonMCI, and PD-MCI groups (Table 5). Table 6 showed that the optimal cut-off score of the MMSE was ≤ 26 (*p* < 0.001).

**TABLE 4:** Demographics and clinical characteristics in study groups.

	HC	All PD	PD-nonMCI	PD-MCI	PDD	*p* ^a^	*p* ^b^	*post hoc*

	Mean (SD)	Mean (SD)	Mean (SD)	Mean (SD)	Mean (SD)			
Sample size	82	84	46	25	13	-	-	-
Age, y	64.89 (7.30)	65.79 (8.70)	62.74 (6.63)	67.84 (8.43)	72.62 (11.06)	0.474	<0.001	HC < PDD; PD-nonMCI < PD-MCI = PDD
Male (%)	28%	67%	67%	76%	46%	<0.001	<0.001	HC < PD-nonMCI = PD-MCI; PD-nonMCI = PD-MCI = PDD
Education, y	13.59 (3.30)	11.83 (4.43)	13.11 (3.16)	10.86 (5.13)	9.15 (5.46)	0.004	<0.001	HC = PD-nonMCI > PDD; HC > PD-MCI
Disease duration, y	-	5.87 (4.42)	5.14 (3.96)	6.79 (4.27)	6.72 (5.94)	-	0.052	-
Hoehn–Yahr stage	-	2.05 (0.65)	1.87 (0.50)	0.96 (0.46)	2.92 (0.79)	-	<0.001	PD-nonMCI = PD-MCI < PDD
LED	-	572.5 (376.78)	650.66 (464.94)	478.57 (206.50)	481.82 (208.89)	-	0.168	-
**MDS-UPDRS**								
part I	-	5.82 (5.55)	3.91 (3.16)	7.50 (5.91)	11.11 (9.08)	-	<0.001	PD-nonMCI < PD-MCI = PDD
part II	-	7.23 (6.84)	4.63 (3.30)	8.79 (7.54)	16.33 (9.61)	-	0.001	PD-nonMCI < PD-MCI < PDD
part III	-	35.27 (9.72)	32.16 (9.04)	36.50 (7.85)	47.56 (7.28)	-	0.001	PD-nonMCI = PD-MCI < PDD
ADLs	99.94 (0.55)	96.07 (8.11)	99.02 (4.03)	96.80 (7.62)	84.23 (9.54)	<0.001	<0.001	HC = PD-nonMCI > PD-MCI > PDD
original PDSFS	55.04 (8.53)	50.15 (9.08)	52.48 (7.55)	49.04 (9.52)	44.08 (10.61)	<0.001	<0.001	HC = PD-nonMCI > PDD; HC > PD-MCI = PDD
FHS	24.39 (3.44)	23.01 (4.96)	24.50 (3.47)	22.20 (4.97)	19.31 (5.20)	0.031	<0.001	HC = PD-nonMCI = PD-MCI; HC = PD-nonMCI > PDD
IRRL	21.55 (4.09)	22.29 (4.55)	22.76 (4.15)	21.80 (4.63)	21.54 (5.84)	0.275	0.487	-
SB	9.10 (3.45)	4.94 (2.89)	5.22 (2.64)	5.32 (3.16)	3.23 (2.77)	<0.001	<0.001	HC > PD-nonMCI = PD-MCI = PDD

*HC, healthy controls; PD, Parkinson’s disease; PD-nonMCI, PD without mild cognitive impairment; PD-MCI, Parkinson’s disease with mild cognitive impairment; PDD, Parkinson’s disease with dementia; SD, standard deviation; LED, levodopa equivalent dosage; MDS-UPDRS, movement disorder society-sponsored revision of the unified Parkinson’s disease rating scale; ADLs, activity of daily living scale; original PDSFS, Parkinson’s disease social functioning scale; FHS, Family Life, Hobbies, and Self-Care; IRRL, Interpersonal Relationship and Recreational Leisure; SB, Social Bond.*

*^a^Comparison between HC and all PD with the Bonferroni correction (p < 0.006).*

*^b^Comparison between HC, PD-nonMCI, PD-MCI, and PDD with the Bonferroni correction (p < 0.0035).*

**TABLE 5:** Neuropsychological performance in study groups.

	HC	All PD	PD-nonMCI	PD-MCI	PDD	*p* ^a^	*p* ^b^	*post hoc*
	**Mean (SD)**	**Mean (SD)**	**Mean (SD)**	**Mean (SD)**	**Mean (SD)**			
**Global cognition**								
MMSE	27.39 (2.08)	25.52 (3.54)	27.46 (1.85)	24.56 (3.28)	20.54 (2.99)	<0.001	<0.001	HC = PD-nonMCI > PD-MCI = PDD
original MoCA	26.16 (3.06)	21.89 (4.64)	24.52 (2.61)	20.64 (4.08)	15.00 (2.94)	<0.001	<0.001	HC = PD-nonMCI > PD-MCI > PDD
**Executive function**								
MCST-C	4.88 (1.58)	3.94 (1.67)	4.18 (1.50)	3.51 (3.94)	-	0.005	0.006	HC = PD-nonMCI; HC > PD-MCI
CTT-B	111.61 (38.99)	156.75 (92.87)	121.41 (22.67)	221.76 (131.89)	-	0.001	<0.001	HC = PD-nonMCI < PD-MCI
**Attention**								
Digit span	13.00 (2.57)	11.37 (2.66)	12.15 (2.45)	9.92 (2.45)	-	<0.001	<0.001	HC = PD-nonMCI > PD-MCI
CTT-A	54.95 (16.19)	72.25 (37.33)	62.63 (11.55)	95.64 (55.49)	-	0.001	<0.001	HC = PD-nonMCI < PD-MCI
**Language**								
Similarities	12.52 (2.46)	10.48 (2.87)	11.02 (2.61)	9.48 (3.10)	-	<0.001	<0.001	HC = PD-nonMCI; HC > PD-MCI
Category fluency	41.87 (7.85)	32.54 (7.98)	33.91 (6.98)	30.00 (9.17)	-	<0.001	<0.001	HC > PD-nonMCI = PD-MCI
**Memory**								
LM-II	12.95 (2.44)	9.79 (3.93)	10.98 (3.78)	7.60 (3.25)	-	<0.001	<0.001	HC > PD-nonMCI > PD-MCI
VR-II	11.10 (2.45)	9.69 (2.87)	10.70 (2.79)	7.84 (1.99)	-	<0.001	<0.001	HC = PD-nonMCI > PD-MCI
**Visuospatial function**								
Block design	11.17 (2.72)	9.68 (2.77)	10.43 (2.48)	8.28 (2.76)	-	<0.001	<0.001	HC = PD-nonMCI > PD-MCI
Matrix reasoning	12.71 (3.17)	1.07 (3.15)	11.89 (2.87)	9.56 (3.14)	-	<0.001	<0.001	HC = PD-nonMCI > PD-MCI

*please see Tables 3, 4 and MMSE, mini-mental state examination; original MoCA, Montreal Cognitive Assessment.*

*^a^Comparison between HC and all PD (ANCOVA adjusting for sex and education) with the Bonferroni correction (p < 0.004).*

*^b^Comparison between HC, PD-nonMCI, PD-MCI, and PDD (ANCOVA was adjusting for age, sex, and education) with the Bonferroni correction (p < 0.004).*

**TABLE 6:** The usage of the total score of MMSE and its subtests to detect patients with PD-MCI.

Item (maximums score)	AUC	*p*	Cutoff	Sensitivity	Specificity	Youden index
Total score of MMSE (30)	0.802	<0.001	≤ 26	0.680	0.813	0.463
Orientation (10)	0.634	0.063	<9	0.520	0.674	0.194
Time (5)	0.689	0.009	<5	0.560	0.761	0.321
Place (5)	0.500	1.000	<3	0.080	0.978	0.058
Registration (8)	0.689	0.009	<8	0.640	0.696	0.336
Name 3 objects (3)	0.509	0.885	<3	0.040	0.978	0.018
Serial 7’s (5)	0.655	0.032	<5	0.600	0.717	0.317
Recall (3)	0.624	0.085	<2	0.320	0.913	0.233
Language (5)	0.577	0.284	<5	0.240	0.913	0.153
Naming (2)	0.500	1.000	≤ 1	0.000	1.000	0.000
Repetition (1)	0.487	0.861	<1	0.040	0.935	-0.025
Reading (1)	0.560	0.406	<1	0.120	1.000	0.120
Writing (1)	0.578	0.279	<1	0.200	0.917	0.157
Following command (3)	0.654	0.033	<3	0.400	0.913	0.313
Copy design (1)	0.587	0.226	<1	0.240	0.935	0.175

*MMSE, mini-mental state examination; AUC, the area under the curve.*

*Bonferroni correction was applied with a p value < 0.003.*

### Generation of the Simplified Montreal Cognitive Assessment and Simplified Parkinson’s Disease Social Functioning Scale Scores

The relationships between each variable (e.g., neuropsychological domains, factors of social function, and demographic variables) were evaluated by Spearman correlation and excluded highly correlated variables (Spearman correlation coefficient > 0.7) (Table 7). We used logistic regression to identify the most predictive models of MoCA and PDSFS (Table 8). When comparing the AUC of the three MoCA models, the third model had the maximum AUC (0.767 and 0.844). It was named the simplified MoCA and had the most effective combination to detect PD-MCI and PDD. The simplified MoCA contains “visuospatial/executive, memory, and orientation domains,” and the total score is 16. Regarding the PDSFS Models, the first model had the maximum AUC (0.652 and 0.672) and was named the simplified PDSFS, which successfully detected PD-MCI and PDD. The simplified PDSFS includes “Family Life, Hobbies, and Self-Care,” and the total score is 27. The total score of the combination of these two tests is 43.

**TABLE 7:** The Spearman rho correlation between the MoCA domains, the PDSFS factors, and demographic variables.

	Age	Sex	Education	VE	Naming	Memory	Attention	Language	Abstraction	Orientation	FHS	IRRL
Sex	0.06	-										
Education	–0.16*	0.08	-									
**original MoCA**												
Visuospatial/executive	–0.27**	–0.02	0.43***	-								
Naming	–0.30***	0.21**	0.29***	0.39***	-							
Memory	–0.22**	–0.27**	0.26**	0.31***	0.12	-						
Attention	–0.25**	0.02	0.39***	0.30***	0.31***	0.34***	-					
Language	–0.26**	–0.22**	0.32***	0.39***	0.21**	0.45***	0.36***	-				
Abstraction	–0.18*	–0.07	0.35***	0.39***	0.26**	0.35***	0.36***	0.42***	-			
Orientation	–0.27**	–0.07	0.20**	0.39***	0.37***	0.25**	0.31***	0.30***	0.24**	-		
**original PDSFS**												
FHS	–0.09	–0.13	–0.02	0.15	0.03	0.13	0.01	0.19*	0.15	0.14	-	
IRRL	0.11	–0.03	–0.17*	–0.02	–0.08	–0.04	–0.13	–0.01	–0.04	–0.03	0.47***	-
Social Bond	–0.07	–0.24*	0.16	0.30***	0.14	0.34***	0.11	0.21**	0.22**	0.21**	–0.28***	0.17*

*original MoCA, Montreal Cognitive Assessment; VE, Visuospatial/executive; original PDSFS, Parkinson’s disease social functioning scale; FHS, Family Life, Hobbies, and Self-Care; IRRL, Interpersonal Relationship and Recreational Leisure.*

*p < 0.05; **p < 0.01; ***p < 0.001.

**TABLE 8:** The best predictive model of the MoCA and the PDSFS for PD-MCI and PDD by using the logistic regression with the backward stepwise.

Model and neurocognitive domains (maximums score)		B	SE	*p*	Exp (B)	AUC^a^	AUC^b^
**Model of original MoCA**							
Model 1	Memory domain (5)	–0.511	0.163	0.002	0.600	0.696	0.574
Model 2	Visuospatial/executive domain (5)	–1.244	0.459	0.016	0.007	0.695	0.838
	Concentration domain (6)	0.544	0.253	0.031	1.723		
Model 3	Visuospatial/executive domain (5)	–0.640	0.255	0.012	0.527	0.767	0.844
	Memory domain (5)	–0.522	0.195	0.007	0.593		
	Orientation domain (6)	0.479	0.186	0.010	1.615		
**Model of original PDSFS**							
Model 1	Family Life, Hobbies, and Self-Care (27)	–0.207	0.059	<0.001	0.813	0.652	0.672
Model 2	Social Bond (14)	–0.550	0.223	0.014	0.577	0.549	0.702

*original MoCA, Montreal Cognitive Assessment; original PDSFS, Parkinson’s disease social functioning scale.*

*^a^Discrimination PD-MCI from PD-nonMCI.*

*^b^Discrimination PDD from PD-MCI.*

### Using Simplified Versions of the Montreal Cognitive Assessment and Parkinson’s Disease Social Functioning Scale and a Combination of These Two Tests to Detect Parkinson’s Disease With Mild Cognitive Impairment and Parkinson’s Disease With Dementia (Table 9)

#### Discriminating Parkinson’s Disease With Mild Cognitive Impairment From Parkinson’s Disease Without Mild Cognitive Impairment

The AUCs for the simplified MoCA, simplified PDSFS, and a combination of these two tests were 0.767, 0.652, and 0.790, respectively. The optimal cut-off score for combining these two tests was ≤ 35 (sensitivity: 0.760; specificity: 0.717, *p* < 0.001).

**TABLE 9:** The psychometric properties of the MoCA and PDSFS to detect PD-MCI and PDD in this study and relevant studies.

Item (maximums score)	PD-nonMCI vs. PD-MCI						PD-MCI vs. PDD					
	AUC	*p*	Cutoff	Sensitivity	Specificity	Youden index	AUC	*p*	Cutoff	Sensitivity	Specificity	Youden index
original MoCA (30)	0.801	<0.001	≤ 23	0.760	0.717	0.477	0.869	<0.001	≤ 18	0.846	0.800	0.646
original PDSFS (68)	0.603	0.152	≤ 56	0.760	0.413	0.173	0.652	0.128	≤ 44	0.538	0.760	0.298
simplified MoCA (16)	0.767	<0.001	≤ 9	0.440	0.935	0.375	0.846	0.001	≤ 7	0.769	0.840	0.609
simplified PDSFS (27)	0.652	0.036	≤ 25	0.720	0.565	0.285	0.672	0.085	<23	0.769	0.680	0.449
Combination (43)	0.790	<0.001	≤ 35	0.760	0.717	0.477	0.794	0.003	≤ 30	0.846	0.640	0.486
**Relevant studies**												
MoCA algorithm (30)	0.733	0.001	≤ 23	0.640	0.696	0.336	0.835	0.001	≤ 20	1.000	0.600	0.600
MoCA-5-min (12)	0.767	<0.001	≤ 7	0.800	0.630	0.430	0.714	0.032	≤ 6	0.846	0.560	0.406
Short MoCA-Czech (16)	0.754	<0.001	≤ 8	0.520	0.913	0.433	0.751	0.012	≤ 7	0.846	0.600	0.446
Short MoCA-US (16)	0.763	<0.001	≤ 9	0.560	0.870	0.390	0.751	0.012	≤ 8	0.846	0.560	0.406

*AUC, area under the curve; PD-nonMCI, Parkinson’s disease without mild cognitive impairment; PD-MCI, Parkinson’s disease with mild cognitive impairment; PDD, Parkinson’s disease with dementia; original MoCA, Montreal Cognitive Assessment; original PDSFS, Parkinson’s disease social functioning scale.*

*Bonferroni correction was applied with a p value < 0.005.*

#### Discriminating Parkinson’s Disease With Dementia From Parkinson’s Disease With Mild Cognitive Impairment

The AUCs for the simplified MoCA, simplified PDSFS, and a combination of these two tests were 0.846, 0.672, and 0.794, respectively. The optimal cut-off score for combining these two tests was 30 (sensitivity: 0.846; specificity: 0.640, *p* = 0.003).

### Comparison of the Discriminative Power of the Original and Simplified Version (Table 9)

#### Comparison Between the Original and Simplified Version of Montreal Cognitive Assessment and Parkinson’s Disease Social Functioning Scale

Both the original MoCA and the simplified MoCA can be used to distinguish PD-nonMCI from PD-MCI and distinguish PD-MCI from PDD (*p* < 0.001). Moreover, Fengler (2016) developed a scoring algorithm for the original MoCA. Sophie’s MoCA algorithm was designed to improve the ability to discriminate PD-MCI. Compared with Sophie’s MoCA algorithm, the AUC for the original MoCA was better than Sophie’s MoCA algorithm in discriminating between PD-nonMCI and PD-MCI (AUC: 0.801 > 0.733). When distinguishing PD-MCI from PDD, the AUCs for the original MoCA and Sophie’s MoCA algorithm were equivalent (AUC: 0.869 and 0.835). In addition, both versions of the PDSFS could neither distinguish PD-nonMCI from PD-MCI nor distinguish PD-MCI from PDD.

#### Comparison Between the Simplified Version of Montreal Cognitive Assessment and Relevant Studies

The AUCs for the simplified MoCA, MoCA-5 min, short MoCA-Czech, and short MoCA-US were equivalent in discriminating between PD-nonMCI and PD-MCI. The relevant methods (e.g., MoCA-5-min, short MoCA-Czech, short MoCA-US) cannot be used to distinguish PD-MCI and PDD (*p* > 0.005). Our simplified MoCA (*p* = 0.001) can be used to distinguish PD-MCI and PDD.

## Discussion

In the present study, we considered both cognitive and social functions to determine the diagnosis of patients with PD. First, we simplified the original versions of MoCA and PDSFS and provided the optimal cut-off score to detect cognitive and social dysfunctions in patients with PD. We suggest that combining the simplified versions of MoCA and PDSFS is an effective and helpful tool to detect PD-MCI and PDD in a clinical setting.

### Using Mini-Mental State Examination and Its Subtest to Detect Parkinson’s Disease With Mild Cognitive Impairment

Mini-Mental State Examination is one of the most commonly used screening tools for detecting PDD (Hoops et al., 2009; Dalrymple-Alford et al., 2010; Ozdilek and Kenangil, 2014) or PD-MCI (Hoops et al., 2009; Dalrymple-Alford et al., 2010; Biundo et al., 2014; Ozdilek and Kenangil, 2014; Yu et al., 2020). We found that the optimal cutoff for MMSE to detect PD-MCI was 26, which is consistent with a previous research report (Ozdilek and Kenangil, 2014). Many studies have proposed various cut-off scores (Hoops et al., 2009; Dalrymple-Alford et al., 2010; Biundo et al., 2014; Yu et al., 2020). The various PD-MCI criteria may explain conflicting results (Hoops et al., 2009; Ozdilek and Kenangil, 2014), different standard deviations to define MCI (Dalrymple-Alford et al., 2010; Biundo et al., 2014), and various grouping methods (Federico et al., 2015; Yu et al., 2020). This study used strict diagnostic criteria (MDS Level II) (Litvan et al., 2012) as the basis for grouping. Moreover, we found no single subtest of MMSE is suitable for detecting PD-MCI. Therefore, we do not recommend using the single subtest of MMSE to detect PD-MCI.

### Using the Original Versions of Montreal Cognitive Assessment and Original Versions of Parkinson’s Disease Social Functioning Scale to Detect Parkinson’s Disease With Mild Cognitive Impairment and Parkinson’s Disease With Dementia

The optimal original MoCA cut-off scores for detecting PD-MCI and PDD were 23 and 18. Our findings are similar to those of previous studies (Uysal-Cantürk et al., 2018; Badrkhahan et al., 2019); however, our results are inconsistent with those of other studies (Hoops et al., 2009; Dalrymple-Alford et al., 2010; Marras et al., 2013; Biundo et al., 2014; Kandiah et al., 2014; Ozdilek and Kenangil, 2014; Federico et al., 2015; Xu et al., 2015; Mazancova et al., 2020; Yu et al., 2020). The cultural background/language (Hoops et al., 2009; Biundo et al., 2014; Ozdilek and Kenangil, 2014; Federico et al., 2015; Uysal-Cantürk et al., 2018; Badrkhahan et al., 2019; Mazancova et al., 2020), grouping method (e.g., various diagnostic criteria) (Hoops et al., 2009; Chen et al., 2013; Marras et al., 2013; Xu et al., 2015), the various standard deviation of tests to define impairment (e.g., –1, –1.5, –2 SD below normative data) (Hoops et al., 2009; Dalrymple-Alford et al., 2010; Biundo et al., 2014; Ozdilek and Kenangil, 2014; Yu et al., 2020), could explain this discrepancy.

Cultural backgrounds and languages may cause variation in findings. Bezdicek et al. (2020) found that language factors could explain 26.1% of the variance of MoCA-Czech, implying that language and cultural differences should be considered when developing culturally specific versions. The construct of an item is ambiguous due to a lack of cultural equivalent, where there is unfamiliarity with testing, or when the related skill does not exist within a cultural schema (O’Driscoll and Shaikh, 2017). Moreover, different diagnostic criteria and SD of the neuropsychological tests to define PD-MCI would influence the sensitivity to detect PD-MCI. Evidence showed that liberal standards (–1 SD) have the highest sensitivity and are the most suitable screening standards (Goldman et al., 2013). This study used the MDS PD-MCI criteria and liberal standards to recruit the target group.

We found that the original PDSFS alone (Su et al., 2020) is unsuitable for detecting PD-MCI and PDD. However, we found that the PD patients have a lower overall social function (i.e., a total score of PDSFS) than healthy aging. Su et al. (2020) found that patients with PDD had the worst social function, and PD patients without dementia had lower social function than healthy older adults. Given the heterogeneity of the PD population without dementia, we further divided them into two groups (i.e., PD-MCI and PD-nonMCI) in this study. We found that PD-nonMCI patients have a similar overall social function as the healthy aging, and PD-MCI patients have a similar overall social function as the PDD. In addition, we found that the healthy aging’s “Social Bound” was better than that of PD patients; moreover, the “Social Bound” of PD-nonMCI and PD-MCI patients were comparable to those of PDD patients. This indicates that the social connection of PD without dementia (i.e., PD-nonMCI and PD-MCI) may begin to worsen. Anderson et al. (2013) demonstrated that patients with PD-MCI had difficulties in spontaneous metalizing, which may negatively impact interpersonal relationships. Their result indicates that the social connection of the PD population may begin to worsen. Our study revealed that PD-nonMCI, PD-MCI, and PDD groups showed different profiles of social functioning impairments. More research and further research are warranted.

### Using the Simplified Versions of Montreal Cognitive Assessment and the Simplified Versions of Parkinson’s Disease Social Functioning Scale to Detect Parkinson’s Disease With Mild Cognitive Impairment and Parkinson’s Disease With Dementia

Our simplified MoCA contains “visuospatial/executive, memory, and orientation domains,” and our simplified PDSFS includes “Family Life, Hobbies, and Self-Care.” The total score of simplified MoCA and simplified PDSFS is 16 and 27, respectively. We suggest that the patients’ cognitive performance be classified as PD-MCI when the simplified MoCA score is ≤ 9. Furthermore, the patients would be categorised as PDD when the simplified MoCA score is 7. However, the simplified PDSFS alone cannot detect PD-MCI and PDD.

Three MoCA short versions were commonly applied in the PD population (Dong et al., 2015; Roalf et al., 2016; Bezdicek et al., 2020); unfortunately, only the Czech (Bezdicek et al., 2020) and English (Roalf et al., 2016) versions are available. Previous studies about the cross-cultural applicability of MoCA showed that languages, cultural factors, lifestyles, and the education systems would differ between countries, and the content was required to be tailored more accurately in further revision (O’Driscoll and Shaikh, 2017; Bezdicek et al., 2020). Here, we developed a simplified MoCA version and found that the discrimination ability of our simplified MoCA was equivalent to that of the original MoCA in detecting PD-MCI and PDD. Compared with short MoCA-Czech (Bezdicek et al., 2020), short MoCA-US (Roalf et al., 2016), and MoCA-5-min protocol (Dong et al., 2015), our simplified MoCA contains executive/visuospatial domains but does not include concentration, language, and abstraction domains (Table 2). We also found that all the relevant short versions can be used to detect PD-MCI; nevertheless, the discrimination of our simplified MoCA is higher than that of the MoCA algorithm, the short MoCA-Czech, the short MoCA-US, and equal to that of the MoCA-5-min protocol. In addition, our simplified MoCA and MoCA algorithm can be used to detect PDD and our simplified MoCA has higher discrimination than the MoCA algorithm. The other relevant short versions (e.g., the short MoCA-Czech, the short MoCA-US, and the MoCA-5-min protocol) are not suitable for detecting PDD.

We found that the short form of the PDSFS is not suitable for detecting PD-MCI and PDD. Nevertheless, comparing the four groups in the “Family Life, Hobbies, and Self-Care” score, the score of the non-demented PD patients (i.e., PD-nonMCI group and PD-MCI group) were similar to healthy aging and better than PDD patients. The small number of people in the PD-MCI group may have contributed to the statistical insignificance. Although there was no statistical significance in the mean scores of “Family Life, Hobbies, and Self-Care” score between healthy aging and non-demented PD group (i.e., PD-nonMCI and PD-MCI groups), we found that the scores in the PD-MCI group were intermediate between the PD-nonMCI group and PDD group; that is, PD-MCI patients may have worse “Family Life, Hobbies, and Self-Care” function than deficit PD-nonMCI patients and better than PDD patients. The PD-MCI patients might have difficulty dealing with primary personal needs (e.g., food preparation, medication, and self-cleaning). Previous studies indicate that PD-MCI patients receive significantly lower medicine and financial management (Pirogovsky et al., 2014); accordingly, these items are included in our simplified PDSFS. Moreover, Becker et al. (2020) suggested that handling finances and managing transportation are impaired in patients with PD-MCI but not in PD patients without dementia. Therefore, we suggest that PD-MCI and PDD may impair social function, especially in personal care and interpersonal interaction.

### The Combination of These Two Tests Is Helpful in Detecting Parkinson’s Disease With Mild Cognitive Impairment and Parkinson’s Disease With Dementia

We considered both cognitive and social functions together to detect PD-MCI or PDD. To the best of our knowledge, no study has evaluated the two factors simultaneously. Our findings suggested that the combination of the two simplified tests had the unique advantage of providing cognitive and social functioning information to detect PD-MCI and PDD, and was more efficient to administer than the original MoCA (Nasreddine et al., 2005), original PDSFS (Su et al., 2020), our simplified MoCA, our simplified PDSFS, and other relevant short versions. The patients can be classified into PD-MCI and PDD categories when the total score of the combination of the two tests is ≤ 35 and ≤ 30, respectively.

We used the Wilcoxon test to compare the discriminative power of original MoCA, original PDSFS, simplified MoCA, simplified PDSFS, and the combination of the two simplified tests. The results demonstrate that the power of the combination of the two simplified tests is similar to that of the original MoCA (comparing AUCs of PD-nonMCI vs. PD-MCI: *z* = -0.131, *p* = 0.895; comparing AUCs of PD-MCI vs. PDD: *z* = -0.695, *p* = 0.486) and simplified MoCA (comparing AUCs of PD-nonMCI vs. PD-MCI: *z* = 0.266, *p* = 0.791; comparing AUCs of PD-MCI vs. PDD: *z* = -0.468, *p* = 0.640). Our findings and previous evidence (Anderson et al., 2013; Pirogovsky et al., 2014; Perepezko et al., 2019; Becker et al., 2020; Su et al., 2020) showed that patients with PDD have deteriorated social functioning, and the decline may happen in the earlier stage of the disease (i.e., PD-nonMCI and PD-MCI). We found that the vulnerable aspect was especially in “Family Life, Hobbies, and Self-Care” and “Social Bound.” The cognitive assessment alone is not enough to know whether the patients experience any social functioning impairment, especially subtle social function changes. Based on clinical needs to take sufficient information on social and cognitive functions, we suggest using the combination of the two tests to detect PD-MCI and PDD.

## Limitations

During the COVID-19 pandemic, the government recommended reducing unnecessary social activities, and people preferred staying at home. Therefore, some candidates’ willingness to join the study was affected, making participant recruitment more difficult. Moreover, we used a more rigorous method (i.e., the Level II criteria) to define PD-MCI to accurately classify and increase the reliability and validation of our results. Some patients could not complete the comprehensive assessment due to fatigue or time constraints. As a result, it was difficult to recruit participants, and the sample size was small. Second, we applied the MDS PD-MCI level II diagnostic criteria to diagnose PD-MCI patients; however, the rigorous criteria led to a small number of participants in this group. Third, the AUC of the combination version was similar to that of the original MoCA; however, the time required to complete the combination version is shorter than the original MoCA, which is beneficial to clinical use. In addition, clinicians can obtain detailed information about the patient’s cognitive and social functions through the combination version, which is helpful for diagnosis and subsequent intervention. Last but not least, to the best of our knowledge, this is the first study to examine the detection of MCI or dementia by combining cognitive and social function tests. More research is needed in the future, especially considering cultural backgrounds and languages (Bezdicek et al., 2020) and specific motor functions (Chuang et al., 2022) that may affect patients’ cognitive and social functioning.

## Conclusion

Our simplified MoCA can be used to detect PD-MCI and PDD (cut-off scores: 9 and 7) efficiently. Moreover, the patients can be classified into the PD-MCI and PDD categories when the total score of the combination of the two tests is 35 and 30, respectively. Given the cruciality of social functioning and the limitation of cognitive screening tools, combining the two tests will help evaluate cognitive and social functions efficiently and help the physician decide on further intervention. This is the first study to develop an instrument that considers both social and cognitive functions to the best of our knowledge. More study is needed to validate our findings and focus on exploring the patients’ social functioning in the disease course.

## Nomenclature

### Resource Identification Initiative

“A New Instrument Combines Cognitive and Social Functioning Items for Detecting Mild Cognitive Impairment and Dementia in Parkinson’s Disease (National Cheng Kung University; Tainan; Taiwan) RRID:SCR_000980.”

## Data Availability Statement

The raw data supporting the conclusions of this article will be made available by the authors, without undue reservation.

## Ethics Statement

The studies involving human participants were reviewed and approved by National Cheng Kung University Hospital and Kaohsiung Medical University Hospital. Written informed consent to participate in this study was provided by the participants’ legal guardian/next of kin.

## Author Contributions

All authors made substantial contributions to the conception and design of the work, acquisition, analysis and interpretation of data, drafted the work and revised it, gave final approval of the completed version, and agreed to be accountable for all aspects of the work.

## Conflict of Interest

The authors declare that the research was conducted in the absence of any commercial or financial relationships that could be construed as a potential conflict of interest.

## Publisher’s Note

All claims expressed in this article are solely those of the authors and do not necessarily represent those of their affiliated organizations, or those of the publisher, the editors and the reviewers. Any product that may be evaluated in this article, or claim that may be made by its manufacturer, is not guaranteed or endorsed by the publisher.

## References

[B1] Aarsland D. Andersen K. Larsen J. P. Lolk A. (2003). Prevalence and characteristics of dementia in Parkinson disease. *Arch. Neurol.* 60:387. 10.1001/archneur.60.3.387 12633150

[B2] Aarsland D. Kurz M. W. (2010). The epidemiology of dementia associated with Parkinson’s disease. *Brain Pathol.* 20 633–639. 10.1111/j.1750-3639.2009.00369.x 20522088 PMC8094858

[B3] Anderson R. J. Simpson A. C. Channon S. Samuel M. Brown R. G. (2013). Social problem solving, social cognition, and mild cognitive impairment in Parkinson’s disease. *Behav. Neurosci.* 127:184. 10.1037/a0030250 23067384

[B4] Armstrong M. J. Okun M. S. (2020). Diagnosis and treatment of parkinson disease: a review. *JAMA* 323 548–560. 10.1001/jama.2019.22360 32044947

[B5] Badrkhahan S. Z. Sikaroodi H. Sharifi F. Kouti L. Noroozian M. (2019). Validity and reliability of the Persian version of the Montreal Cognitive Assessment (MoCA-P) scale among subjects with Parkinson’s disease. *Appl. Neuropsychol. Adult* 27 431–439. 10.1080/23279095.2019.1565762 30821505

[B6] Becker S. Bäumer A. Maetzler W. Nussbaum S. Timmers M. Van Nueten L. (2020). Assessment of cognitive-driven activity of daily living impairment in non-demented Parkinson’s patients. *J. Neuropsychol.* 14 69–84. 10.1111/jnp.12173 30320954

[B7] Bennett D. A. Schneider J. A. Tang Y. Arnold S. E. Wilson R. S. (2006). The effect of social networks on the relation between Alzheimer’s disease pathology and level of cognitive function in old people: a longitudinal cohort study. *Lancet Neurol.* 5 406–412. 10.1016/s1474-4422(06)70417-3 16632311

[B8] Bettencourt B. Sheldon K. (2001). Social roles as mechanism for psychological need satisfaction within social groups. *J. Pers. Soc. Psychol.* 81 1131–1143. 11761313

[B9] Bezdicek O. Èervenková M. Moore T. M. Stepankova Georgi H. Sulc Z. Wolk D. A. (2020). Determining a short form montreal cognitive assessment (s-MoCA) czech version: validity in mild cognitive impairment Parkinson’s disease and cross-cultural comparison. *Assessment* 27 1960–1970. 10.1177/1073191118778896 29929376 PMC6274600

[B10] Biundo R. Weis L. Facchini S. Formento-Dojot P. Vallelunga A. Pilleri M. (2014). Cognitive profiling of Parkinson disease patients with mild cognitive impairment and dementia. *Parkinson. Relat. Disord.* 20 394–399. 10.1016/j.parkreldis.2014.01.009 24495708

[B11] Chen J. H. (2002). *Wechsler Adult Intelligence Scale- (Chinese Version): Administration and Scoring Manual*, 3rd Edn. Taipei: Chinese Behavioral Science Corporation.

[B12] Chen L. Yu C. Fu X. Liu W. Hua P. Zhang N. (2013). Using the montreal cognitive assessment scale to screen for dementia in Chinese patients with Parkinson’s disease. *Shanghai Arch. Psychiatry* 25 296–305. 10.3969/j.issn.1002-0829.2013.05.005 24991170 PMC4054575

[B13] Chen M.-L. Tan C.-H. Su H.-C. Sung P.-S. Chien C.-Y. Yu R.-L. (2021). The impact of sex on the neurocognitive functions of patients with Parkinson’s disease. *Brain Sci.* 11:1331. 10.3390/brainsci11101331 34679396 PMC8533932

[B14] Chuang Y. H. Tan C. H. Su H. C. Chien C. Y. Sung P. S. Lee T. L. (2022). Hypomimia may influence the facial emotion recognition ability in patients with Parkinson’s disease. *J. Parkinsons Dis.* 12 185–197. 10.3233/jpd-212830 34569974

[B15] Dalrymple-Alford J. C. MacAskill M. R. Nakas C. T. Livingston L. Graham C. Crucian G. P. (2010). The MoCA: well-suited screen for cognitive impairment in Parkinson disease. *Neurology* 75 1717–1725. 10.1212/WNL.0b013e3181fc29c9 21060094

[B16] D’Elia L. Satz P. Uchiyama C. L. White T. (1996). *Color Trails Test.* Odessa, FL: PAR.

[B17] Dong Y. Koay W. I. Yeo L. L. L. Chen C. L.-H. Xu J. Seet R. C. S. (2015). Rapid screening for cognitive impairment in Parkinson’s disease: a pilot study. *Parkinsons Dis.* 2015 1–6. 10.1155/2015/348063 26713170 PMC4680112

[B18] Dubois B. Burn D. Goetz C. Aarsland D. Brown R. G. Broe G. A. (2007). Diagnostic procedures for Parkinson’s disease dementia: recommendations from the movement disorder society task force. *Mov. Disord.* 22 2314–2324. 10.1002/mds.21844 18098298

[B19] Emre M. Aarsland D. Brown R. Burn D. J. Duyckaerts C. Mizuno Y. (2007). Clinical diagnostic criteria for dementia associated with Parkinson’s disease. *Mov. Disord.* 22 1689–1707. 10.1002/mds.21507 17542011

[B20] Fankhauser S. Forstmeier S. Maercker A. Luppa M. Luck T. Riedel-Heller S. G. (2015). Risk of dementia in older adults with low versus high occupation-based motivational processes:differential impact of frequency and proximity of social network. *J. Geriatr. Psychiatry Neurol.* 28 126–135. 10.1177/0891988714554706 25431449

[B21] Federico A. Maier A. Vianello G. Mapelli D. Trentin M. Zanette G. (2015). Screening for mild cognitive impairment in Parkinson’s disease: comparison of the italian versions of three neuropsychological tests. *Parkinsons Dis.* 2015 1–10. 10.1155/2015/681976 26634171 PMC4655066

[B22] Fengler S. (2016). Screening for cognitive impairment in Parkinson’s disease: improving the diagnostic utility of the MoCA through subtest weighting. *PLoS One* 11:e0159318. 10.1371/journal.pone.0159318 27437705 PMC4954721

[B23] Folstein M. F. Folstein S. E. McHugh P. R. (1975). “Mini-mental state”: a practical method for grading the cognitive state of patients for the clinician. *J. Psychiatr. Res.* 12 189–198. 1202204 10.1016/0022-3956(75)90026-6

[B24] Goldman J. G. Holden S. Bernard B. Ouyang B. Goetz C. G. Stebbins G. T. (2013). Defining optimal cutoff scores for cognitive impairment using Movement Disorder Society Task Force criteria for mild cognitive impairment in Parkinson’s disease. *Mov. Disord.* 28 1972–1979. 10.1002/mds.25655 24123267 PMC4164432

[B25] Goldman J. G. Sieg E. (2020). Cognitive impairment and dementia in Parkinson disease. *Clin. Geriatr. Med.* 36 365–377. 32222308 10.1016/j.cger.2020.01.001

[B26] Hanley J. A. McNeil B. J. (1982). The meaning and use of the area under a receiver operating characteristic (ROC) curve. *Radiology* 143 29–36. 10.1148/radiology.143.1.7063747 7063747

[B27] Hoops S. Nazem S. Siderowf A. D. Duda J. E. Xie S. X. Stern M. B. (2009). Validity of the MoCA and MMSE in the detection of MCI and dementia in Parkinson disease. *Neurology* 73 1738–1745. 10.1212/WNL.0b013e3181c34b47 19933974 PMC2788810

[B28] Hua M. S. Chang B. S. Lin K. N. Yang J. M. Lu S. R. Chen S. Y. (2005). *Wechsler Memory Scale-Third Edition (Chinese Version): Administration and Scoring Manual.* Taipei: Chinese Behavioral Science Corporation.

[B29] Hua M.-S. Chang S.-H. Chen S.-T. (1997). Factor structure and age effects with an aphasia test battery in normal Taiwanese adults. *Neuropsychology* 11:156. 10.1037//0894-4105.11.1.156 9055279

[B30] Kandiah N. Zhang A. Cenina A. R. Au W. L. Nadkarni N. Tan L. C. (2014). Montreal Cognitive Assessment for the screening and prediction of cognitive decline in early Parkinson’s disease. *Parkinson. Relat. Disord.* 20 1145–1148. 10.1016/j.parkreldis.2014.08.002 25176439

[B31] Katz S. (1983). Assessing self-maintenance: activities of daily living, mobility, and instrumental activities of daily living. *J. Am. Geriatr. Soc.* 31 721–727. 10.1111/j.1532-5415.1983.tb03391.x 6418786

[B32] Litvan I. Aarsland D. Adler C. H. Goldman J. G. Kulisevsky J. Mollenhauer B. (2011). MDS task force on mild cognitive impairment in Parkinson’s disease: critical review of PD-MCI. *Mov. Disord.* 26 1814–1824. 10.1002/mds.23823 21661055 PMC3181006

[B33] Litvan I. Goldman J. G. Tröster A. I. Schmand B. A. Weintraub D. Petersen R. C. (2012). Diagnostic criteria for mild cognitive impairment in Parkinson’s disease: Movement Disorder Society Task Force guidelines. *Mov. Disord.* 27 349–356. 10.1002/mds.24893 22275317 PMC3641655

[B34] Liu W.-M. Lin R.-J. Yu R.-L. Tai C.-H. Lin C.-H. Wu R.-M. (2015). The impact of nonmotor symptoms on quality of life in patients with Parkinson’s disease in Taiwan. *Neuropsychiatr. Dis. Treat.* 11 2865–2873. 10.2147/NDT.S88968 26635475 PMC4646598

[B35] Marras C. Armstrong M. J. Meaney C. A. Fox S. Rothberg B. Reginold W. (2013). Measuring mild cognitive impairment in patients with Parkinson’s disease. *Mov. Disord.* 28 626–633. 10.1002/mds.25426 23520128 PMC4524474

[B36] Mazancova A. F. Rùžièka E. Jech R. Bezdicek O. (2020). Test the best: classification accuracies of four cognitive rating scales for Parkinson’s disease mild cognitive impairment. *Arch. Clin. Neuropsychol.* 35 1069–1077. 10.1093/arclin/acaa039 32681175

[B37] Nasreddine Z. S. Phillips N. A. Bedirian V. Charbonneau S. Whitehead V. Collin I. (2005). The Montreal Cognitive Assessment, MoCA: a brief screening tool for mild cognitive impairment. *J. Am. Geriatr. Soc.* 53 695–699. 10.1111/j.1532-5415.2005.53221.x 15817019

[B38] Nelson H. E. (1976). A modified card sorting test sensitive to frontal lobe defects. *Cortex* 12 313–324. 10.1016/s0010-9452(76)80035-4 1009768

[B39] O’Driscoll C. Shaikh M. (2017). Cross-cultural applicability of the montreal cognitive assessment (MoCA): a systematic review. *J. Alzheimers Dis.* 58 789–801. 10.3233/JAD-161042 28482634

[B40] Ozdilek B. Kenangil G. (2014). Validation of the Turkish version of the montreal cognitive assessment scale (MoCA-TR) in patients with Parkinson’s disease. *Clin. Neuropsychol.* 28 333–343. 10.1080/13854046.2014.881554 24528299

[B41] Perepezko K. Hinkle J. T. Shepard M. D. Fischer N. Broen M. P. G. Leentjens A. F. G. (2019). Social role functioning in Parkinson’s disease: a mixed-methods systematic review. *Int. J. Geriatr. Psychiatry* 34 1128–1138. 10.1002/gps.5137 31069845 PMC6949188

[B42] Petersen R. C. Doody R. Kurz A. Mohs R. C. Morris J. C. Rabins P. V. (2001). Current concepts in mild cognitive impairment. *Arch. Neurol.* 58 1985–1992. 11735772 10.1001/archneur.58.12.1985

[B43] Pinto T. C. Machado L. Bulgacov T. M. Rodrigues-Júnior A. L. Costa M. L. Ximenes R. C. (2019). Is the Montreal Cognitive Assessment (MoCA) screening superior to the Mini-Mental State Examination (MMSE) in the detection of mild cognitive impairment (MCI) and Alzheimer’s Disease (AD) in the elderly. *Int. Psychogeriatr.* 31 491–504. 10.1017/S1041610218001370 30426911

[B44] Pirogovsky E. Schiehser D. M. Obtera K. M. Burke M. M. Lessig S. L. Song D. D. (2014). Instrumental activities of daily living are impaired in Parkinson’s disease patients with mild cognitive impairment. *Neuropsychology* 28 229–237. 10.1037/neu0000045 24417192

[B45] Pringsheim T. Jette N. Frolkis A. Steeves T. D. (2014). The prevalence of Parkinson’s disease: a systematic review and meta-analysis. *Mov. Disord.* 29 1583–1590. 24976103 10.1002/mds.25945

[B46] Roalf D. R. Moore T. M. Wolk D. A. Arnold S. E. Mechanic-Hamilton D. Rick J. (2016). Defining and validating a short form Montreal Cognitive Assessment (s-MoCA) for use in neurodegenerative disease. *J. Neurol. Neurosurg. Psychiatry* 87 1303–1310. 10.1136/jnnp-2015-312723 27071646 PMC5061594

[B47] Robben S. H. M. Sleegers M. J. M. Dautzenberg P. L. J. Van Bergen F. S. Ter Bruggen J.-P. Rikkert M. G. M. O. (2010). Pilot study of a three-step diagnostic pathway for young and old patients with Parkinson’s disease dementia: screen, test and then diagnose. *Int. J. Geriatr. Psychiatry* 25 258–265. 10.1002/gps.2331 19582760

[B48] Saredakis D. Collins-Praino L. E. Gutteridge D. S. Stephan B. C. Keage H. A. (2019). Conversion to MCI and dementia in Parkinson’s disease: a systematic review and meta-analysis. *Parkinson. Relat. Disord.* 65 20–31. 10.1016/j.parkreldis.2019.04.020 31109727

[B49] Schapira A. H. V. Chaudhuri K. R. Jenner P. (2017). Non-motor features of Parkinson disease. *Nat. Rev. Neurosci.* 18 435–450. 10.1038/nrn.2017.62 28592904

[B50] Su F.-T. Tai C.-H. Tan C.-H. Hwang W.-J. Yu R.-L. (2020). The development of the social functioning scale for patients with Parkinson’s disease. *J. Parkinsons Dis.* 10 1143–1151. 10.3233/JPD-201930 32444559

[B51] Uysal-Cantürk P. Hanaðası H. A. Bilgiç B. Gürvit H. Emre M. (2018). An assessment of Movement Disorder Society Task Force diagnostic criteria for mild cognitive impairment in Parkinson’s disease. *Eur. J. Neurol.* 25 148–153. 10.1111/ene.13467 28941002

[B52] Winblad B. Palmer K. Kivipelto M. Jelic V. Fratiglioni L. Wahlund L. O. (2004). Mild cognitive impairment–beyond controversies, towards a consensus: report of the International Working Group on Mild Cognitive Impairment. *J. Intern. Med.* 256 240–246. 10.1111/j.1365-2796.2004.01380.x 15324367

[B53] Xu S. L. G. Zhu I. H. Li Y. G. (2015). The research of using Montreal cognitive assessment to evaluate mild cognitive impairment in patients with Parkinson’s disease. *Chinese J. Rehabil. Med.* 3:256.

[B54] Yu R.-L. Chen P. S. Tu S.-C. Tsao W.-C. Tan C.-H. (2018). Emotion-specific affective theory of mind impairment in Parkinson’s disease. *Sci. Rep.* 8:16043. 10.1038/s41598-018-33988-6 30375420 PMC6207749

[B55] Yu R. L. Lee W. J. Li J. Y. Chang Y. Y. Chen C. C. Lin J. J. (2020). Evaluating mild cognitive dysfunction in patients with Parkinson’s disease in clinical practice in Taiwan. *Sci. Rep.* 10;1014. 10.1038/s41598-020-58042-2 31974411 PMC6978523

[B56] Yu R.-L. Tan C.-H. Wu R.-M. (2015a). The impact of nocturnal disturbances on daily quality of life in patients with Parkinson’s disease. *Neuropsychiatr. Dis. Treat.* 11 2005–2012. 10.2147/NDT.S85483 26273203 PMC4532217

[B57] Yu R.-L. Tan C.-H. Wu Y.-R. Wu R.-M. Chiu M.-J. Hua M.-S. (2015b). Memory for gist and detail information in patients with Parkinson’s disease. *BMJ Open* 5:e009795. 10.1136/bmjopen-2015-009795 26589429 PMC4663425

[B58] Yu R.-L. Wu R. M. (2013a). An exploration of relationships among emotional decoding ability, motor symptoms and non-motor experiences in non-demented Parkinson’s disease. *Mov. Disord.* 28:S105.

[B59] Yu R.-L. Wu R.-M. (2013b). Social brain dysfunctions in patients with Parkinson’s disease: a review of theory of mind studies. *Transl. Neurodegener.* 2:7. 10.1186/2047-9158-2-7 23537376 PMC3621839

[B60] Yu R.-L. Wu R.-M. Chan A. Y. Y. Mok V. Wu Y.-R. Tilley B. C. (2017). Cross-Cultural Differences of the Non-Motor Symptoms Studied by the traditional chinese version of the international parkinson and movement disorder society-unified Parkinson’s disease rating scale. *Mov. Disord. Clin. Pract.* 4 68–77. 10.1002/mdc3.12349 28345011 PMC5365089

[B61] Yu R.-L. Wu R.-M. Tai C.-H. Lin C.-H. Cheng T.-W. Hua M.-S. (2012a). Neuropsychological profile in patients with early stage of Parkinson’s disease in Taiwan. *Parkinson. Relat. Disord.* 18 1067–1072. 10.1016/j.parkreldis.2012.06.002 22749792

[B62] Yu R. L. Wu R. M. Chiu M. J. Tai C. H. Lin C. H. Hua M. S. (2012b). Advanced theory of mind in patients at early stage of Parkinson’s disease. *Parkinson. Relat. Disord.* 18 21–24. 10.1016/j.parkreldis.2011.08.003 21868278

[B63] Yu R. L. Wu R. M. Tai C. H. Lin C. H. Hua M. S. (2010). Feeling-of-knowing in episodic memory in patients with Parkinson’s disease with various motor symptoms. *Mov. Disord.* 25 1034–1039. 10.1002/mds.23017 20131392

